# Quality of Pharmaceutical Care in Surgical Patients

**DOI:** 10.1371/journal.pone.0101573

**Published:** 2014-07-09

**Authors:** Monica de Boer, Maya A. Ramrattan, Eveline B. Boeker, Paul F. M. Kuks, Marja A. Boermeester, Loraine Lie-A-Huen

**Affiliations:** 1 Department of Hospital Pharmacy, Academic Medical Centre, Amsterdam, The Netherlands; 2 Department of Surgery, Academic Medical Centre, Amsterdam, The Netherlands; University of South Florida, United States of America

## Abstract

**Background:**

Surgical patients are at risk for preventable adverse drug events (ADEs) during hospitalization. Usually, preventable ADEs are measured as an outcome parameter of quality of pharmaceutical care. However, process measures such as QIs are more efficient to assess the quality of care and provide more information about potential quality improvements.

**Objective:**

To assess the quality of pharmaceutical care of medication-related processes in surgical wards with quality indicators, in order to detect targets for quality improvements.

**Methods:**

For this observational cohort study, quality indicators were composed, validated, tested, and applied on a surgical cohort. Three surgical wards of an academic hospital in the Netherlands (Academic Medical Centre, Amsterdam) participated. Consecutive elective surgical patients with a hospital stay longer than 48 hours were included from April until June 2009. To assess the quality of pharmaceutical care, the set of quality indicators was applied to 252 medical records of surgical patients.

**Results:**

Thirty-four quality indicators were composed and tested on acceptability and content- and face-validity. The selected 28 candidate quality indicators were tested for feasibility and ‘sensitivity to change’. This resulted in a final set of 27 quality indicators, of which inter-rater agreements were calculated (kappa 0.92 for eligibility, 0.74 for pass-rate). The quality of pharmaceutical care was assessed in 252 surgical patients. Nearly half of the surgical patients passed the quality indicators for pharmaceutical care (overall pass rate 49.8%). Improvements should be predominantly targeted to medication care related processes in surgical patients with gastro-intestinal problems (domain pass rate 29.4%).

**Conclusions:**

This quality indicator set can be used to measure quality of pharmaceutical care and detect targets for quality improvements. With these results medication safety in surgical patients can be enhanced.

## Introduction

The quality of health care systems is an important area of concern. In hospitals, one of every 150 admitted patients dies as a consequence of adverse events. [1] Almost 60% of these in-hospital events are associated with surgical care. [1] In the past years, there has been an increased awareness in patient safety in surgery. Therefore, several effective strategies are developed to measure and improve patient safety in the surgical processes, such as the use of Surgical Safety checklists. [2]–[5]

Medication-related adverse events also occur in surgical patients. [6], [7] These events are known as adverse drug events (ADEs). [8] The incidence of ADEs in surgical patients varies between 2.1–27.7 ADEs per 100 admissions of which 15.3%–53.6% of the ADEs are preventable. [6] In a recent study, the occurrence of preventable ADEs was measured to be 4.2 per 100 admissions in the surgical population in hospitals using computerized physician order entry (CPOE) systems with clinical decision support. [7] This indicates that medication-related events are still a problem in hospitals.

Preventable ADEs are often used as an outcome measure for improvements in the quality of in-hospital pharmaceutical care. [9]–[11] These ADEs are usually measured retrospectively by screening patient records on potential ADEs using, for example, a so-called trigger tool method. [8], An expert panel then assesses the causality of found triggers with medications to determine the actual presence of ADEs. This approach seems effective [14] and widely accepted in several patient populations. [12], [13], [15]–[18] However, it is a time consuming method and depending on the substantial contribution of the expert panel.

Another approach to assess improvements in the quality of in-hospital care is the use of process measures, such as quality indicators (QIs). [19], [20] Process measures elucidate suboptimal care in the process, and are influenced directly by implementations of quality improvements. [21], [22] Furthermore, these measures are easier to recognize than outcome measures and are more efficient in assessing the quality of care. [21], [22] QIs are explicitly defined and measurable items that refer to processes of care, but can also refer to structures or outcomes of care. [20] Focusing on the processes of care, a commonly used example of these measures is the quality indicator set to assess the quality of pharmacologic care of vulnerable elderly patients (ACOVE). [23], [24] Also, a set of quality indicators has been developed to assess the care of elderly surgical patients. [25] However, only few of these indicators are directly related to the medication processes on the surgical ward.

Due to the above mentioned reasons, we composed and tested a set of quality indicators specifically for the medication use in the surgical population in addition to the outcome assessment of ADEs. [7] Furthermore, we assessed the quality of pharmaceutical care of several medication-related processes using QIs on surgical wards in order to detect targets improvements.

## Methods

### Setting and population

In this observational cohort study three surgical wards of an academic hospital in the Netherlands (Academic Medical Centre, Amsterdam) participated. Consecutive elective surgical patients with a hospital stay longer than 48 hours were included from April until June 2009. Patients were excluded when transferred from another ward within the same hospital or transferred between hospitals. Only the first elective admission of each patient was assessed during the observation period. The surgical wards contained mainly gastro-intestinal and vascular surgery patients.

### Selection and composition of QI set

To compose a representative set of QIs for medication-related processes on surgical wards, several frequently occurring problems in the surgical population for which medication is used (medication care related problems), were selected by an expert panel. This expert panel consisted of a consultant surgeon (MB) and hospital pharmacist (PK). The problems were defined into domains in which the QIs were to be classified: pain, infection, thrombosis, gastro-intestinal problem, delirium and ‘other’ problem. The documentation and handovers of medication-related information between care providers (communication related problem) was also selected as a frequently occurring problem in the surgical population.

Several inclusion criteria were noted to select eligible QIs for the surgical QI set:[26]

aimed at medication-related processes on surgical wards;
applicable to a surgical population and administrative data; and
able to measure changes in medication-related processes (due to interventions by physicians or hospital pharmacists).

The selection of QIs was predominantly based on existing sets of QIs. [23]–[25], [27] After selecting the QIs, these were adapted to the surgical population incorporating national and local surgical care guidelines. Expert opinion of the consultant surgeon and hospital pharmacist was used during this consensus based selection and composition process of QIs. We choose to define the quality indicators according to the ‘IF…THEN’-principle from ACOVE [28] with explicitly defined denominator and numerator.

### Testing of QI set

Several steps were performed to determine if the composed QIs were eligible to apply on the surgical population. Firstly, the expert panel individually reviewed the developed QIs on acceptability, i.e. relevance and appropriateness for the surgical population, and on content validity and face validity, such as phrasing and comprehensibility. [20], [27], [29], [30] They scored these aspects on a 9-point Likert scale. If a score of five or below was assessed by the expert panel, we indicated this test as fail and excluded the quality indicator. If disagreement occurred between the experts, consensus based conclusions were made on their testing results leading to inclusion or exclusion of the indicator.

Next, the feasibility of the remaining QIs was tested in 50 randomly selected test patients admitted to the surgical wards between December 2008 and February 2009 to assess the overall usability. Different aspects of feasibility were scored by two individual reviewers, i.e. the availability of data and the clinical burden to collect data on a 9-point Likert scale, the occurrence of eligibility (e.g. the number of patients eligible for the indicator; >1%), and the timeframe necessary to apply the QI set to a patient. [20], [29] A timeframe of one hour is considered acceptable to apply a QI set on a single patient. [23], [25], [31] Furthermore, the ‘sensitivity to change’ was calculated using the indicators pass rates (100% minus pass rate, e.g. the percentage of eligible patients meeting the QI). [20] Since no literature was available on a cutoff point, we considered a quality indicator to be ‘sensitive to change’ when a pass rate of 90% or less was reached. In the last step, the inter-rater agreement was assessed to obtain information about the reliability of the composed QI set. [20], [27], [31] Inter-rater reliability was assessed by two independent reviewers on 50 test patients. The agreement between reviewers on eligibility and on pass rates of the quality indicators on the eligible patients, was calculated by use of Cohen's kappa (κ) statistic [32] in SPSS version 18.0 (SPSS Inc., Chicago, IL). Values for κ between 0.41 and 0.60 were considered to reflect moderate agreement, values between 0.61 and 0.80 substantial agreement, and values >0.81 almost perfect agreement. [33] Also, percentages of agreement on eligibility and pass rates of the quality indicators in this patient population were calculated between the reviewers.

### Assessment of quality of pharmaceutical care

The composed and tested QI set was retrospectively applied on the medical records of 252 surgical patients, admitted between March and June 2009. The number of eligible patients was determined, as well as the pass rates of the quality indicator in the total patient population. The pass rate of an indicator represents the percentage of eligible patients that met that particular quality indicator. [23], [24], [31] If the QI could not be evaluated due to insufficient data, this was scored as unknown. Mean pass rates were calculated for each domain of QIs. Evaluation of the pass rates will detect targets for quality improvements in this population.

### Comparison of QIs with ADEs

ADEs are outcome measures and QIs are process measures. A preventable ADE can be the result of a failing medication process. Depending on the content of QIs, ADEs can therefore have a direct relationship with the QIs. However, if no direct relationship is present, then QIs and ADEs can have a complementary value in measuring the quality of pharmaceutical care. To study and describe the interaction and complementary value of the QIs and ADEs, each ADE and preventable ADE was linked to the corresponding QI domain, if possible. The method to detect and assess ADEs and preventable ADEs in this population is previously described by De Boer et al. [13]

### Ethics and informed consent

After evaluation by the Medical Ethics Committee at the Academic Medical Centre Amsterdam, it was decided that this study was exempt from ethical approval, because this study did not meet the criteria for Medical Scientific Research with Humans under the Dutch Law. Informed consent was therefore not required. Patient records and information was anonymized prior to analysis.

## Results

### Selection and composition of QI set

**Figure 1:**
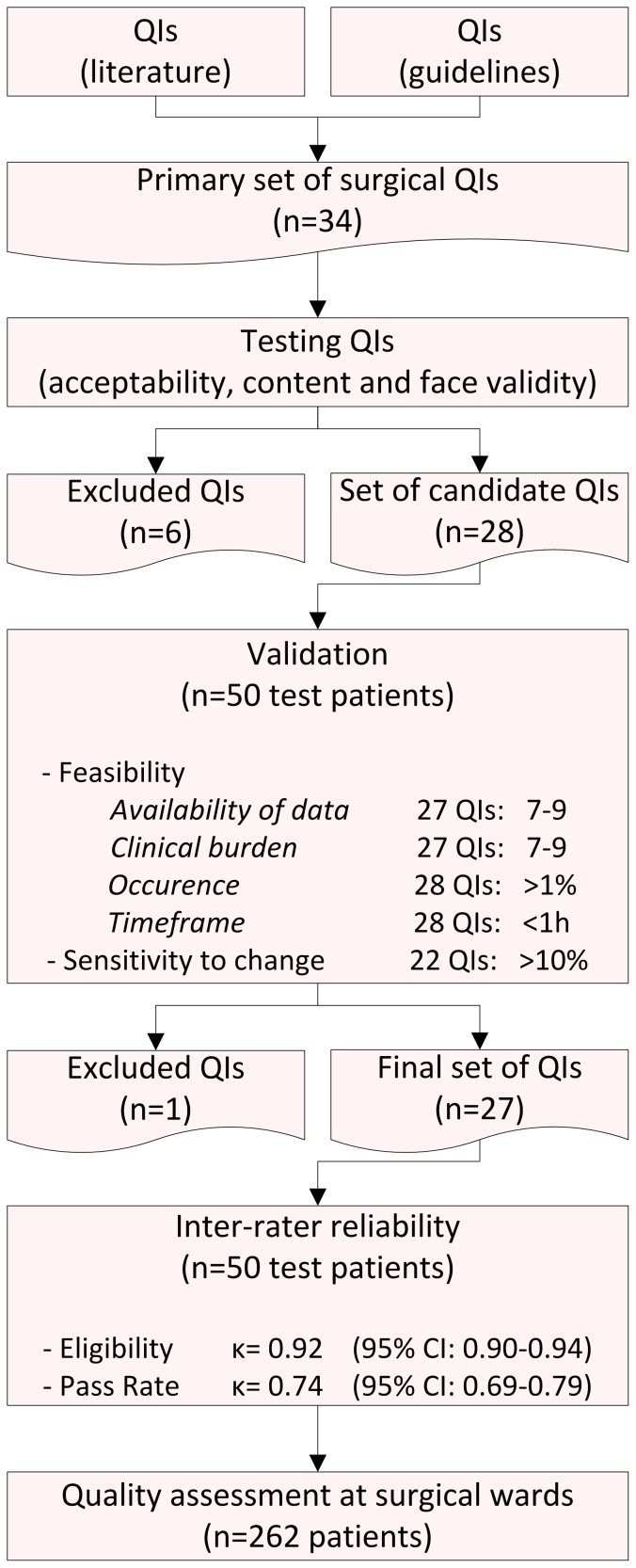
Flowchart composition and testing of surgical QI set. *Excluded QIs are reported in table 1 and 2.

The QIs were divided in domains representing medication care related problems, e.g. pain, infection, thrombosis, gastrointestinal problem, delirium, and ‘other’ problem, and in communication related problems, e.g. the domain documentation and discharge. Based on QIs from existing sets and national guidelines, 34 quality indicators were primarily composed for the pharmaceutical care in the surgical population. A flowchart of the composition and testing of the surgical QI set is depicted in figure 1.

### Testing of QI set

The expert panel tested the QIs on acceptability, content validity and face validity. Six QI were excluded based on these tests (table 1). The 28 candidate QIs were tested for feasibility and sensitivity to change on 50 test patients (table 2). The availability of data and the clinical burden was scored 7–9 on the 9-point Likert scale for 27 indicators, demonstrating that these QIs were acceptable for application in the surgical population. One indicator scored poor on feasibility (3 for availability of data, 6 for clinical burden). All candidate indicators occurred more than 1% in the test population. Furthermore, the complete set of 28 QIs was scored on patients’ records within one hour with an average time of 49 minutes per patient. In addition, table 1 shows the average times necessary to score each indicator. The ‘sensitivity to change’ was scored more than 10% for 22 indicators and 0–8% for the six remaining indicators. In four of these QIs the number of eligible patients was very low (2–6% of 50 patients), indicating an unreliable result on ‘sensitivity to change’. In the remaining two QIs the sensitivity to change was 4% and 8%, indicating a high pass rate score, thus hardly any improvement of the quality of care was possible. Based on the test population of 50 patients, it cannot be definitively concluded that these QIs will not be useful to assess the quality of pharmaceutical care. Furthermore, the feasibility was good for these six QIs. As a result, it was decided to exclude only one indicator (no. 28 in table 2) from the set due to poor feasibility. This QI was defined as: “If a specification of the discharge medication of the patient is included in the discharge letter, THEN there should be none of the following discrepancies with the medication used as an outpatient UNLESS it is noted in the patients' records: differences in names of used medication, dosages, route of administration, formulation”. The 27 selected QIs are displayed in table 3.

**Table 1:** Excluded QIs.

Quality Indicator	Acceptability (1–9)^a^		Content validity (1–9)	Face validity (1–9)	
	Relevance	Appropriateness		Phrasing	Comprehensibility
IF a surgical patient receives pain medication and has changes in pain conditions, THEN the pain medication must be adjusted according to the patient's pain condition	9	5	5	5	5
IF epidural analgesics are stopped on a surgical patient, THEN the patient must receive a booster dose of Tramadol before the epidural pain treatment is stopped AND the pain medication is switched to Tramadol	3	4	5	8	9
IF a surgical patient is prescribed antibiotic therapy, THEN the date of start and stop and/or duration of the therapy should be noted in the patient's records	9	5	9	7	5
IF a surgical patient uses an oral or intravenous opiate for more than 3 days, THEN a laxative should be prescribed only when the doses of the opiate has the potential for constipation	5	3	5	3	5
IF a surgical patient is admitted to the surgical care unit for a planned surgery, THEN within 6 months prior to the operation a preoperative screening should be performed by the anaesthetist and the results are documented in the patients charts.	7	5	9	9	9
IF a surgical patient is being discharged with changes in his/her medication-profile, THEN the patient discharge instruction includes information about medication	5	9	4	1	1

a 1 = total disagreement, 9 =  total agreement.

**Table 2:** Testing results of QIs.

QI^a^	Feasibility (n = 50)				Sensitivity to change (n = 50) (%)
	Availability of data (1–9)^b^	Clinical burden (1–9)^c^	Occurrence (%)	Timeframe (min)	
**1**	7.9	7.8	68	3.9	38
**2**	8.8	8.7	32	2.1	12
**3**	8.8	8.7	90	2.1	27
**4**	8.9	8.8	26	1.4	54
**5**	8.9	9.0	90	1.1	4
**6**	8.7	6.8	22	2.4	73
**7**	7.8	7.0	8	7.0	25
**8**	8.0	8.3	6	3.0	33
**9**	8.0	8.0	6	2.7	0
**10**	9.0	9.0	6	1.3	100
**11**	8.5	8.5	4	3.5	50
**12**	9.0	9.0	2	2.0	0
**13**	8.9	8.9	98	1.0	8
**14**	8.8	8.8	98	1.0	90
**15**	8.8	8.8	84	1.6	95
**16**	8.8	8.5	48	2.4	21
**17**	8.5	8.5	4	2.5	50
**18**	8.0	8.0	4	4.5	0
**19**	7.5	9.0	4	4.0	50
**20**	8.5	8.0	4	4.5	50
**21**	8.4	8.2	88	3.5	59
**22**	8.0	8.0	6	4.3	67
**23**	8.6	8.8	90	2.2	60
**24**	8.7	8.0	90	3.3	98
**25**	9.0	9.0	100	1.1	58
**26**	8.9	8.9	100	1.0	58
**27**	9.0	9.0	2	1.0	0
**28**	6.7	6.0	40	3.3	90

a The content of QIs 1–27 are displayed in table 3.

b Calculated mean of two reviewers; 1 = total disagreement, 9 =  total agreement.

c Calculated mean of two reviewers; 1 = high clinical burden, 9 = low clinical burden.

**Table 3:** QI set with eligibility and pass rates.

	Quality Indicators (n = 252)	Eligible patients	Pass rate
		*n (%)*	*n (%)* a
	Domain: Pain		Mean^b^ 65.5%
1	IF a surgical patient receives pain medication and has a pain-score of 4 or higher, THEN the pain medication must be adjusted to lower this pain score.	180 (71.4)	73 (40.6)
2a	IF a surgical patient receives a NSAID AND has 1 or more of the following risk factors: previous ulcer, age >70 years, untreated *H. pylori*-infection with presence of ulcer, THEN the patient should receive a proton pump inhibitor or at least 400 mcg misoprostol.	28 (11.1)	24 (85.7)
2b	IF a surgical patient of 60–70 years receives a NSAID AND has 1 or more of the following cumulative risk factors: high dose of NSAID (>DDD), simultaneous use of oral anticoagulants, acetylsalicylic acid, oral corticosteroids, SSRI and/or spironolacton, serious co-morbidity (invalidating rheumatoid arthritis, heart failure and/or diabetes mellitus), THEN the patient should receive a proton pump inhibitor or at least 400 mcg misoprostol.	40 (15.9)	38 (95.0)
	**Domain: Infection**		**Mean 71.0%**
3	IF a surgical patient receives peri-operative antibiotic prophylaxis, THEN the patient should receive the antibiotic prophylaxis within 60 to 15 minutes prior to the incision.	209 (82.9)	145 (69.4)
4	IF the surgical patient receives antibiotic prophylaxis AND the duration of the surgery lasts longer than 4 hours OR there is more than 2 litres of blood loss OR extracorporeal circulation is used, THEN the antibiotic dose should be repeated.	53 (21.0)	23 (43.4)
5	IF a surgical patient receives antibiotic prophylaxis, THEN this should not be given for more than 24 hours after surgery.	209 (82.9)	208 (99.5)
6	IF a surgical patient receives antibiotics i.v. for more than 3 days, THEN this regimen should be switched to oral antibiotics UNLESS the patient is unable to tolerate oral medications OR reasonable doubts on oral efficacy persist.	35 (13.9)	22 (62.9)
7	IF a surgical patient has documented renal insufficiency and is prescribed antibiotics, THEN the prescribed dose of antibiotics should be adjusted according to current guidelines.	11 (4.4)	10 (90.9)
8	IF a surgical patient receives empiric antibiotic treatment and an antibiogram is assessed, THEN the antibiotic treatment should be adjusted according to this antibiogram within 24 hours after it becomes available.	15 (6.0)	9 (60.0)
	**Domain: Thrombosis**		**Mean 48.6%**
9	IF a surgical patient receives acenocoumarol or fenprocoumon prior to the surgery, THEN the vitamin-K-antagonist should be stopped for at least 3 days or respectively 7 days before surgery UNLESS reason of continuation is documented preoperatively.	11 (4.4)	8 (72.7)
10	IF a surgical patient uses a vitamin-K-antagonist prior to the surgery, THEN the INR should be determined not more than 24 hours prior to surgery.	11 (4.4)	5 (45.5)
11	IF a surgical patient restarts vitamin-K-antagonist therapy postoperatively, THEN the INR should be checked within 1 day after restart of the vitamin-K-antagonist.	9 (3.6)	2 (22.2)
12	IF a surgical patient starts vitamin-K-antagonist therapy postoperatively, THEN the INR should be checked within 3 days after start of the vitamin-K-antagonist.	2 (0.8)	1 (50.0)
13	IF a surgical patient receives thrombo-embolism prophylaxis during admission, THEN the patient should have 1 or more of the following risk factors for thrombo-embolism complications: age >60 yr, (morbid) obesities (BMI >30), long-lasting bed rest >7 days, malignity or chemotherapy, thrombosis in anamnesis, heart failure NYHA III-IV, chronic obstructive pulmonary disease (COPD), Inflammatory Bowel Disease (IBD), oral anticonception, pregnancy and childbed, varicosis, thrombophilia.	251 (99.6)	219 (87.3)
14	IF thrombo-embolism prophylaxis is administered to the surgical patient, THEN time of administration of the thrombo-embolism prophylaxis should be according to the current guidelines for: 1. Fraxiparine: at least 12 hours after surgery; 2. Fondaparinux: at least 6 hours after surgery; 3. Unfractionated heparin: at least 8 hours after surgery	246 (97.6)	34 (13.8)
	**Domain: Gastrointestinal problem**		**Mean 29.4%**
15	IF a surgical patient uses an opioid, THEN a laxative should be used simultaneously OR the reason for omitting a laxative should be documented in the medical records.	207 (82.1)	15 (7.2)
16	IF a surgical patient is feeling nauseated and/or is vomiting after surgery, THEN the patient should be treated with metoclopramide or ondansetron/granisetron UNLESS there is a known contraindication.	128 (50.8)	66 (51.6)
	**Domain: Delirium**		**Mean 63.0%**
17	IF a surgical patient uses haloperidol, THEN do NOT prescribe metoclopramide simultaneously OR vice versa.	9 (3.6)	8 (88.9)
18	IF a surgical patient is diagnosed with postoperative delirium and a pharmacological intervention is appropriate, THEN haloperidol treatment should be instituted UNLESS there is a known contraindication.	3 (1.2)	3 (100)
19	IF a surgical patient is diagnosed with delirium, THEN medications that could induce or prolong symptoms of delirium should be adjusted UNLESS the reason for continuation is documented.	3 (1.2)	0 (0)
	**Domain: Other problem**		**Mean 31.4%**
20	IF a surgical patient has a history of cardiac arrhythmias and is newly prescribed QT interval-prolonging medication, THEN this should be prescribed in consultation with a cardiologist.	2 (0.8)	0 (0)
21	IF a surgical patient is undergoing surgery and takes medication as an outpatient, THEN all medications should be continued postoperatively until discharge from the hospital UNLESS it is contraindicated and/or noted in the patients' records.	204 (81.0)	104 (51.0)
22	IF a patient receives controlled-release medication and medication administered through a (naso)gastric or post-pyloric tube, THEN administration of the controlled-release medication through the tube should have been avoided UNLESS administration is possible according to guideline.	37 (14.7)	16 (43.2)
	**Domain: Documentation and discharge**		**Mean 40.0%**
23	IF a surgical patient is admitted to the surgical ward and uses medication as an outpatient, THEN this is documented in the following resources; pre-operative anaesthesiology, surgical medical, nursing, community pharmacy record. This specification includes at least all of the generic or brand names of the medication.	206 (81.7)	98 (47.6)
24	IF the medication is documented in two or more resources, THEN there should be none of the following discrepancies; differences in names of used medication, dosages, route of administration, formulation.	204 (81.0)	9 (4.4)
25	IF a surgical patient is discharged from the surgical ward and a discharge letter is available, THEN this letter should be sent to the outpatients' physician within 5 working days after discharge.	251 (99.6)	110 (43.8)
26	IF a surgical patient is discharged from the surgical ward and a discharge letter is sent to the outpatients' physician, THEN this discharge letter should include a specification of the discharge medication of the patient. Each medication should be identified at least by its generic or brand name.	250 (99.2)	135 (54.0)
27	IF a surgical patient is newly prescribed a vitamin-K-antagonist, THEN enrolment in the outpatient thrombosis service should be documented.	2 (0.8)	1 (50.0)

a % From the number of eligible patients.

b QI 2a and 2b were considered together.

The inter-rater reliability was tested between two reviewers applying the set of 27 QIs on 50 test patients. On eligibility, the κ-value was 0.92 (95% CI 0.90–0.94) indicating almost perfect agreement between the reviewers. The percentage of agreement was 98% on eligibility. On pass rates, the reviewers had a κ-value of 0.74 (95% CI 0.69–0.79), indicating substantial agreement. There was 96.9% agreement between the reviewers.

### Assessment of quality of pharmaceutical care

In total, 262 patients were evaluated for inclusion in this study. The patient characteristics are displayed in table 4.Ten patients were not evaluated with the QI set since their medical records were not completely available.

**Table 4:** Patient characteristics.

Patient characteristic	Subgroup	Patients n (%)
Total n		262
Age in years	17–65	162 (61.8)
	>65	100 (38.2)
Gender	Female	132 (50.4)
	Male	130 (49.6)
BMI (*n = 261*)	<20	22 (8.4)
	20–25	116 (44.4)
	>25	123 (47.1)
Type of Surgery	Gastrointestinal	212 (80.9)
	Vascular	48 (18.3)
	Other	2 (0.8)
ASA classification^a^ (*n = 248)*	I	46 (18.5)
	II	148 (59.7)
	III or IV	54 (21.8)
Co-morbidities (*n = 259*)	No	70 (27.0)
	Yes	189 (73.0)
Cardiovascular co-morbidity^b^	No	138 (53.3)
	Yes	121 (46.7)
COPD/Asthma co-morbidity	No	236 (91.1)
	Yes	23 (8.9)
Diabetes Mellitus co-morbidity	No	224 (86.5)
	Yes	35 (13.5)
Polypharmacy^c^ *(n = 261*)	No	197 (75.5)
	Yes	64 (24.5)
Length of admission (*days median, IQR*)		8 (5–11)
Number of ADE (*n = 76, % of total population*)		85 (33.7)
Number of pADE^d^ (*n = 76, % of total population*)		5 (2.0)

a ASA classification is a score for the fitness of patients prior to surgery.

I: normal healthy, II: mild systemic disease, III: severe systemic function-limiting disease,IV: severe systemic life-threatening disease; ASA score III and IV were considered together, 7 patients had an ASA score of IV.

b includes diseases of the heart and circulation: coronary heart disease, heart failure, congenital heart disease and stroke.

c Polypharmacy: >5 medications on admission.

d preventable ADE.

The results on eligibility and pass rates per QI are shown in table 3. The overall pass rate of pharmaceutical care in surgical patients was 49.8% (range 0%–100%). Mean pass rates were calculated per domain: 65.5% for pain, 71% for infection, 48.6% for thrombosis, 29.4% for gastrointestinal problem, 63% for delirium 31.4% for other problem and 40% for documentation and discharge. Three QIs (12, 20, 27) show less than 1% eligibility indicating that only very few patients met the inclusion criteria for this indicator. In five QIs (14, 15, 19, 20 and 24) a pass rate of less than 20% was found (range 0–13.8%). This indicates that much improvement of pharmaceutical care is possible, especially in the patients on whom QIs 14, 15 and 24 were applicable because large numbers of patients (range 204–246) were eligible for these QIs.

### Comparison of QIs with ADEs

In the population of 252 surgical patients, 85 ADEs (33.7%) and 5 preventable ADEs (2.0%) were found. The ADEs and preventable ADEs were divided, if possible, in the different domains of the QIs (table 5). Most ADEs (70.6%) were caused by medication used for pain. These ADEs were mainly gastrointestinal problems such as nausea/vomiting due to opioids. Other medication (16.5%) accountable for ADEs were for example anesthetics or diuretics, causing mainly hallucinations (due to Ketamine) or electrolyte (potassium) disorders respectively. The preventable ADEs were allergic reactions, hyperkalemia, excessive anticoagulation and electrolyte disorders caused by medication for pain, infections, thrombosis, and ‘other problems’ as categorized in table 5.

**Table 5:** Comparison with ADEs in surgical population.

Domain of QIs	QI Pass rate	ADEs (n = 82)^a^	pADEs (n = 5)
	*Mean %*	*n (%)*	*n (%)*
Pain	65.5	60 (73.2)	1 (20.0)
Infection	71.0	2 (2.4)	1 (20.0)
Thrombosis	48.6	5 (6.1)	1 (20.0)
Gastrointestinal problem	29.4	1 (1.2)	-
Delirium	63.0	-	-
Other problem	31.4	14 (17.1)	2 (40.0)

a Three ADEs were due to two or more different type of medications, these could not be divided into one domain and were therefore excluded from the table.

Compared to the QI pass rates, for example the domain of gastrointestinal problems scored a mean pass rate of only 29.4%, indicating poor quality of pharmaceutical care. However, only one ADE was found in this domain of medication processes, meaning that medication used to treat gastrointestinal problems do not frequently result in harm for the patient. On the other hand, many ADEs (70.6%) occurred due to analgesics (domain pain) and these ADEs were mainly related to gastrointestinal problems. This means that the pharmaceutical care to manage pain and analgesic adverse effects, such as gastrointestinal ADEs, was poor.

## Discussion

In this study, a representative set of valid quality indicators was composed to assess the quality of pharmaceutical care in surgical patients. Based on our results, half of the medication-related processes on the surgical wards need quality improvement.

The QIs set was composed by defining and classifying frequently occurring problems in surgical patients with several criteria, by use of existing QI sets and local surgical guidelines, and by using expert opinion. Several methods have been described to develop indicators, such as the Delphi technique and the RAND/UCLA appropriateness method. [20], [26] Since we only adapted existing QIs, we solely used an iterated consensus method [20] with two experts from the field. They tested acceptability, content validity and face validity for the appropriateness of the developed QIs. Furthermore, feasibility, ‘sensitivity to change’ and reliability were assessed. [20] Until now, McGory et al. [25] were the first to describe quality indicators in elderly patients undergoing surgery. They developed QIs for several perioperative processes, but only 14 of the 91 QIs were related to medication use. These QIs formed the basis for the development of present QI set for surgical patients.

When our QI set was applied to 50 medical records, the inter-rater reliability between two reviewers was almost perfect (κ = 0.92) with regard to eligibility and substantial (κ = 0.72) between two reviewers with regard to pass rate. Similar reliability rates were found by Wierenga et al. [27] when they applied their 87-item QI set on 10 test patients. Good inter-rater reliability results show that the QIs were explicitly defined. Therefore, personal interpretation of the reviewers in applying the QIs was minimal.

Using the QI set in 252 surgical patients resulted in an overall pass rate of 49.8% (range 0%–100%), indicating that the quality of the selected medication-related processes on surgical wards of a Dutch academic centre can be improved substantially. Only Higashi et al. described pass rates while assessing the pharmacological care in elderly using the ACOVE quality indicators. [24] They found an overall pass rate of 74% in several domains; prescribing indicated medications (50%, CI 45–55%), avoiding inappropriate medication (97%, CI 96–98%), education, continuity and documentation (81%, CI 79–84%), and monitoring (64%, CI 60–68%). These QIs are specifically developed for the elderly population, also the defined domains used for the ACOVE QIs as described above, were different compared to our QI set. Therefore, a comparison with our QI pass rates in the surgical population is not valid. Furthermore, no data is available on the quality of the pharmaceutical care assessed with quality indicators in surgical patients. We are, to our knowledge, the first to describe applied QIs for specifically pharmaceutical care in surgical patients.

The mean pass rates of the domains in our population show that much more attention is needed for the medication processes to prevent gastro-intestinal problems (mean pass rate 29.4%) and other problems (mean pass rate 31.4%), such as administering controlled-release medication through a (naso)gastric or post-pyloric tube. The domain documentation and discharge(mean pass rate 40%) shows that registration of the patients' actual medication is liable to many medication errors. For example, 204 patients were eligible for indicator 24 in which, on average, 7.2 discrepancies in medication per patient were found. In 60% of all cases, dosages were registered incorrectly. Such registration errors can lead to medication-related harm and are an important issue of concern in medication safety. [34]

For quality improvements, the QI pass rates should be carefully examined individually. Based on the number of eligible patients, problems can be evaluated and prioritized, and improvements can be implemented. The QIs can be applied again retrospectively after implementation of improvements to assess the actual benefit in the quality of the medication processes or can be applied prospectively during pharmacotherapeutic care. For example, it appeared that adding a laxative to the prescribed opioid therapy to prevent obstipation was not daily practice in surgery or the physicians involved, did not register the reasons for omitting the laxative (pass rate 7.2%). Exploring the reasons for errors in this medication process might lead to improvements that prevent patient harm.

Outcome assessments, such as ADE assessment, constitute a valuable source of information in addition to QIs pass rates. For example, many patients in our surgical population experienced nausea and/or vomiting while being treated with opioids. Although, nausea is a well-known intrinsic side effect of opioids, obstipation due to opioids may lead to nausea as well. ADE assessment helps to distinguish between possible causes of nausea. In our population, the benefit of routine combination of antiemetics (in addition to laxatives) with opioids may very well exceed the risk of side effects from the antiemetics. If this were confirmed in a prospective study, then ADE assessment would have contributed to the development of a new QI.

This study has a number of limitations. First, the present QI set contains a selection of medication processes on surgical wards which were believed to be amenable to quality improvements. However, it appears that three QIs (12, 20, 27) only had 0.8% eligible patients in the total population, so the impact of any quality improvement on the whole population would be small. For these QIs, a larger sample size is necessary to draw conclusions on the quality of pharmaceutical care of these medication processes in surgical patients. It is possible that these medication processes are not applicable for this specific surgical population to assess for quality improvements in clinical practice. Second, our method of assessing the quality of care is retrospective and, therefore, dependent on registration quality and completeness in the patients' medical records. This registration bias may have influenced our findings if physicians and nurses under- or overreport specific clinical information used in our assessment. Third, our QIs were based on current guidelines at the time of development. Standards of care change over time and QIs need to be updated regularly.

Here, a representative and valid QI set was composed to assess quality of pharmaceutical care in surgical patients. On average, half of the medication processes on the surgical wards of a Dutch academic hospital need quality improvement. Also, ADEs are common in this population. Since the QIs are based on literature and guidelines, this QI set can also be used in other surgical settings. Future improvements in the surgical medication processes will lead to increased quality of pharmaceutical care and, therefore, increased medication safety and overall patient safety in surgery.
